# PolypSAM-Open: Mitigating Automation Bias in AI-Assisted Colonoscopy via Open-Set Surgical Artifact Rejection

**DOI:** 10.3390/diagnostics16142226

**Published:** 2026-07-16

**Authors:** Umar Hasan, Shadman Shahriar, Faiyad Hossain, Md Alamgir Hossain, Muhammad Ali Martuza, Sifat Momen

**Affiliations:** 1 Department of Electrical and Computer Engineering, School of Engineering and Physical Sciences, North South University, Dhaka 1229, Bangladesh; umar.hasan@northsouth.edu (U.H.); shadman.shahriar1@northsouth.edu (S.S.); faiyad.hossain@northsouth.edu (F.H.); hossain.md@northsouth.edu (M.A.H.); sifat.momen@northsouth.edu (S.M.); 2 Department of Computer Engineering, College of Computer, Qassim University, Buraydah 51452, Saudi Arabia

**Keywords:** artificial intelligence, intelligent decision support, medical foundation models, open-set recognition, out-of-distribution detection, colorectal polyp segmentation, automation bias, human-AI interaction, clinical safety, computer-aided detection, workflow reliability

## Abstract

**Background:** Intelligent decision support systems for colonoscopy can fail when encountering out-of-distribution surgical instruments such as snares or biopsy forceps, producing false-positive polyp masks that may contribute to automation bias and reduce workflow reliability. This study aimed to develop and evaluate a parameter-efficient framework for open-set-aware polyp segmentation that can reject such anomalous inputs while preserving in-distribution segmentation performance. **Methods:** We propose PolypSAM-Open, which integrates a prototype-based Open-Set Learning (OSL) module with Low-Rank Adaptation (LoRA) in the MedSAM image encoder. The model was trained on Kvasir-SEG using an 85/15 split of authentic polyp images and synthetic high-frequency Gaussian noise to learn a rejection margin. Zero-shot out-of-distribution detection was evaluated on 590 unseen authentic surgical instruments from Kvasir-Instrument. Segmentation and detection performance were compared against a standard MedSAM-LoRA baseline. **Results:** Standard parameter-efficient fine-tuning yielded an OOD AUROC of 0.4263 on authentic surgical instruments. PolypSAM-Open improved zero-shot OOD AUROC to 0.9535 (p<0.001). Despite allocating 15% of training capacity to the synthetic-noise rejection margin, PolypSAM-Open maintained segmentation performance comparable to the standard fine-tuned baseline (Dice 0.9728 versus 0.9723). On ETIS-LaribPolypDB and CVC-ClinicDB, Dice scores were 0.9301 and 0.9386, respectively. The approach remained parameter-efficient, updating 4.48% of total parameters while adding negligible inference latency relative to the underlying MedSAM forward. **Conclusions:** Prototype-based open-set adaptation can substantially improve rejection of unseen surgical artifacts in AI-assisted colonoscopy while preserving high segmentation accuracy. These findings position PolypSAM-Open as a promising strategy for potentially safer decision-support segmentation in endoscopic workflows; prospective clinical validation remains necessary.

## 1. Introduction

Intelligent decision support systems (IDSS) are increasingly used in healthcare, including automated colorectal polyp segmentation during colonoscopy [1]. Foundation models trained on large datasets have broadened representation learning for visual recognition. The Segment Anything Model and its medical derivative, MedSAM [2], have shown strong zero-shot generalization across imaging modalities.

Despite these advancements, deployment in clinical environments introduces safety concerns. Parameter-efficient fine-tuning techniques such as Low-Rank Adaptation (LoRA) [3] enable high-fidelity in-distribution segmentation, but performance can degrade when encountering authentic out-of-distribution (OOD) artifacts [4,5]. During colonoscopy, surgical instruments such as snares or biopsy forceps are common [6]. If these OOD elements are segmented as polyps rather than flagged as unknown, false positives may reduce clinician trust and compromise workflow safety [4,7].

From a human-AI interaction standpoint, this failure mode is particularly insidious. In AI-assisted colonoscopy, endoscopists may exhibit automation bias, an over-reliance on system outputs without independent verification, especially under high cognitive load during long procedures [8]. A model that confidently segments a surgical snare as a polyp produces no visible uncertainty signal to trigger skepticism; the clinician receives a well-formed mask and may act on it. Conversely, a system that reliably flags anomalous inputs as uncertain is intended to support appropriate reliance, returning decision authority to the operator when the AI is operating outside its validated domain.

**Figure 1:**
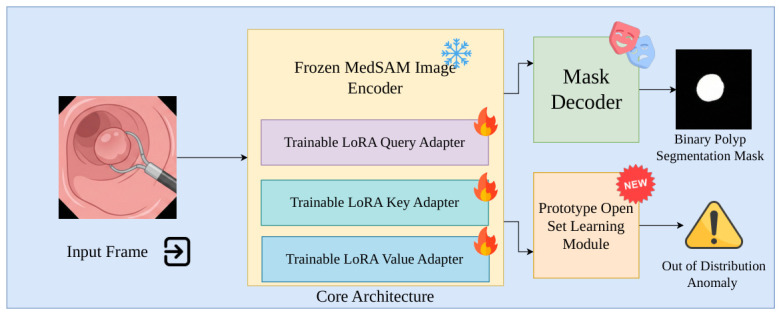
Overview of the PolypSAM-Open framework for open-set-aware polyp segmentation. The input colonoscopy frame (showing both a polyp and a surgical snare artifact) is processed by the frozen, LoRA-adapted MedSAM image encoder. The encoder features feed two parallel heads: the unfrozen SAM decoder for segmentation and the prototype-based Open-Set Learning (OSL) module, which maps features into an embedding space *Z* concentrated around the learned in-distribution prototype *p*. At inference, the Latent Space Distance from *p* yields a novelty score and a safety-flag alert for unseen artifacts such as surgical tools (zero-shot OOD AUROC 0.9535). LoRA: Low-Rank Adaptation; MedSAM: Medical Segment Anything Model; SAM: Segment Anything Model; OSL: Open-Set Learning; OOD: out-of-distribution; AUROC: Area Under the Receiver Operating Characteristic Curve.

To address this gap between predictive performance and clinical reliability, we introduce PolypSAM-Open, a parameter-efficient framework. PolypSAM-Open integrates a prototype-based Open-Set Learning (OSL) module with LoRA in the MedSAM image encoder. As shown in Figure 1, the architecture maps visual patterns into a prototype space to support rejection of unknown artifacts while preserving primary segmentation performance.

To support a true zero-shot OSL setting, training used 85% authentic Kvasir-SEG polyp samples and 15% simulated high-frequency Gaussian noise. The synthetic-noise samples were used to define a latent-space rejection margin. No surgical instrument images were used during training. Zero-shot anomaly detection was then evaluated on 590 unseen authentic surgical instruments from the external Kvasir-Instrument dataset. The architecture remains lightweight for deployment, optimizing 4.48% of total model parameters (4,205,796 of 93,883,440).

Our evaluation shows that open-set-aware adaptation can substantially improve robustness to surgical artifacts while preserving high in-distribution segmentation performance. These findings motivate the proposed framework as a safety-oriented approach for AI-assisted colonoscopy, with detailed experimental results presented in Section 4. The main contributions of this work are as follows:

- We propose PolypSAM-Open, a framework that integrates a prototype-driven Open-Set Learning module with LoRA within MedSAM for open-set-aware polyp segmentation.
- Standard LoRA fine-tuning shows limited OOD discrimination on real surgical tools (AUROC 0.4263), whereas learning a rejection margin from simulated noise improves zero-shot OOD AUROC to 0.9535 using Latent Space Distance.
- We demonstrate that the OSL contrastive margin loss can improve OOD robustness while maintaining segmentation performance comparable to a model trained exclusively on authentic polyp data.
- The proposed adaptation updates 4.48% of network parameters and introduces an inference overhead of approximately 3 ms.

The remainder of this article is organized as follows. Section 2 reviews prior work on medical foundation models, parameter-efficient fine-tuning, and open-set recognition in endoscopy. Section 3 describes the problem formulation, datasets, architecture, and optimization strategy of PolypSAM-Open. Section 4 then presents the quantitative and qualitative results. Section 5 discusses the clinical implications, limitations, and future research directions, and Section 6 concludes the paper.

## 2. Related Work

This section situates PolypSAM-Open within the broader literature most relevant to its design and evaluation. We first review medical foundation models and parameter-efficient fine-tuning strategies for endoscopic image analysis, then discuss the limitations of standard closed-set adaptation in clinically realistic settings. We next examine prior work on OOD detection and the distinction between latent-space and uncertainty-based scoring, and finally summarize the clinical trade-offs, limitations, and translational considerations that motivate the proposed framework.

### 2.1. Medical Foundation Models and Parameter-Efficient Fine-Tuning

The introduction of universal vision models has catalyzed a shift in medical image analysis, with vision transformers and graph-enhanced pipelines setting new standards for gastrointestinal cancer segmentation and complex histopathology analysis [9,10]. MedSAM [2] successfully adapted the Segment Anything Model architecture to the medical domain using over one million image-mask pairs, achieving robust baseline segmentation across various cancer types. However, direct application of these massive models to specialized tasks like colonoscopy requires domain-specific adaptation. Full fine-tuning requires updating all parameters of a very large foundation model. This substantially increases GPU memory consumption, training time, and checkpoint storage, and it raises the risk of overfitting on relatively small medical datasets. Consequently, Parameter-Efficient Fine-Tuning methods have gained traction. LoRA [3], which injects trainable rank decomposition matrices into transformer layers, has proven highly effective. Recent works have highlighted the necessity of this approach. For example, Wu et al. introduced Trans-SAM to transfer SAM to medical tasks using parameter-efficient fine-tuning to prevent catastrophic forgetting [11], and Zhao et al. demonstrated WeakPolyp-SAM for collaborative SAM-driven polyp segmentation [12]. Yang et al. showed that integrating visual prompts with prototype disentanglement enables SAM to effectively overcome domain shifts in specialized segmentation tasks [13]. Similarly, Chen et al. proposed PolypSAMFL, integrating LoRA within a federated learning paradigm for privacy-preserving polyp segmentation [14], while PolySAM-Lite demonstrated that LoRA-based adaptations of SAM can democratize high-performance polyp segmentation for resource-constrained clinical settings [15]. Architectures like Semi-MedSAM [16] and U-SAM2 [17] have also modified foundation model decoders or utilized multi-scale feature fusion to enhance lesion detection. While these adaptations achieve high in-distribution accuracy, they inherently operate under closed-set assumptions and do not address the clinical safety risks posed by unexpected visual artifacts.

### 2.2. Out-of-Distribution Detection in Endoscopy

Recognizing unfamiliar data is a fundamental requirement for the safe deployment of medical AI. In gastroenterology, OOD detection has primarily been explored within the context of wireless capsule endoscopy to identify anomalous frames amidst millions of normal images [18]. Quindós et al. introduced a self-supervised patch-based approach for detecting unseen pathologies in capsule endoscopy [19], while Tan et al. developed EndoOOD to handle undefined categories using uncertainty-aware mixup training [20]. For traditional colonoscopy, Park et al. recently proposed ColonOOD [21], a pipeline integrating OOD detection and uncertainty quantification for polyp classification. In the broader context of foundation models, recent approaches have begun addressing anomaly detection within SAM. Chen et al. proposed SAM-IAD to inject specific anomaly knowledge into SAM via adapters [22], and Peng et al. introduced SAM-LAD for zero-shot anomaly detection using object matching [23]. Despite these efforts, robust OOD detection integrated directly into the dense pixel-level task of polyp segmentation, especially within the architecture of a foundation model, remains largely unexplored.

### 2.3. Open-Set Recognition and Prototype Learning

Open-Set Recognition requires a model to classify known classes while rejecting unknown instances. Pioneering work by Bendale and Boult introduced OpenMax to estimate the probability that an input originates from an unknown class [24]. Subsequent studies have combined vision transformers and open-set learning for unseen biological classes, including a framework for mosquito surveillance [25]. Prototype-based learning has also been used for OSR and dataset-bias mitigation by measuring distances between query samples and learned class embeddings. In the medical domain, Sun et al. introduced SCULPT for OOD detection in whole-slide images [26]; Xu et al. proposed a semi-supervised OSR approach with open mixup and contrastive losses [27]; and Liang et al. proposed dynamic prototype learning for polyp segmentation debiasing [28]. Building on these lines of work, we integrate prototype-based OSL alongside LoRA within a medical foundation model.

### 2.4. Summary of Related Literature

Existing literature successfully addresses medical foundation model adaptation and endoscopic anomaly detection as separate challenges. Current parameter-efficient models excel at segmenting polyps but fail to recognize surgical tools or novel artifacts. Conversely, dedicated OOD frameworks in endoscopy, such as ColonOOD [21], typically operate at the image-level classification level rather than performing dense pixel-level segmentation, the clinically more demanding task that locates and delineates the lesion boundary within the frame. PolypSAM-Open bridges this gap by providing a unified, parameter-efficient architecture that embeds explicit open-set robustness directly into the dense segmentation pipeline of a medical foundation model, delivering both a precise boundary mask and a real-time safety flag from a single forward pass. Table 1 summarizes this distinction relative to existing methods.

## 3. Methods

**Figure 2:**
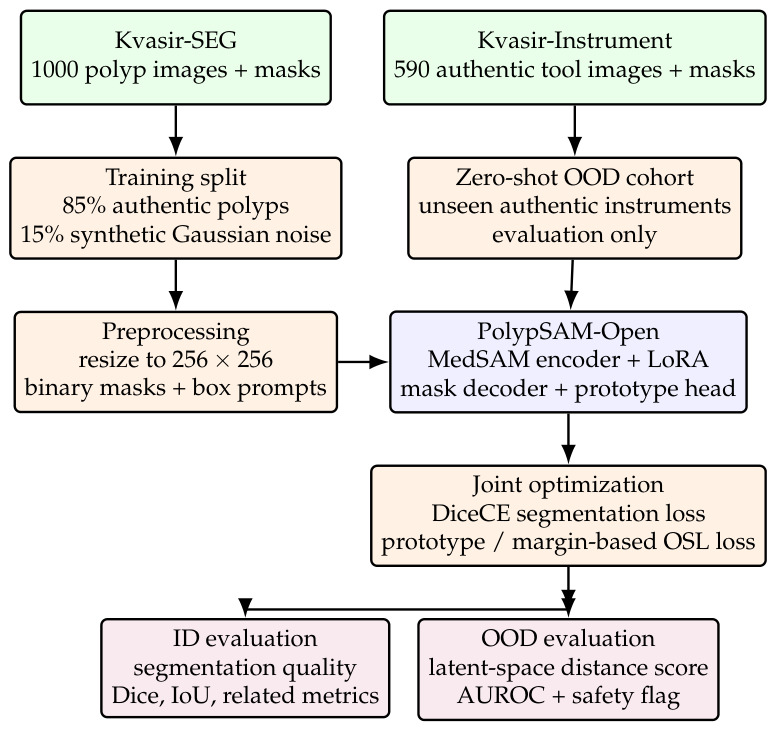
Detailed study workflow for PolypSAM-Open. Kvasir-SEG provides the in-distribution training data, split into authentic polyp samples and synthetic Gaussian-noise samples to shape the latent geometry; Kvasir-Instrument is reserved strictly for zero-shot OOD evaluation. After shared preprocessing and prompt generation, the data pass through the framework, which combines LoRA-adapted MedSAM components with a prototype-based Open-Set Learning head. Training jointly optimizes segmentation fidelity and anomaly separation, followed by separate in-distribution segmentation and out-of-distribution safety evaluation using latent-space distance. ID: in-distribution; OOD: out-of-distribution; LoRA: Low-Rank Adaptation; MedSAM: Medical Segment Anything Model; OSL: Open-Set Learning; AUROC: Area Under the Receiver Operating Characteristic Curve; IoU: Intersection over Union.

This section describes the methodological foundation of PolypSAM-Open. We first formalize the open-set polyp segmentation problem, then describe the datasets and preprocessing pipeline used for in-distribution training and zero-shot OOD evaluation. A procedural overview of the full study workflow is provided in Figure 2. Next, we detail the architecture of the proposed framework, including the integration of LoRA with the prototype-based Open-Set Learning module, and conclude by presenting the training objectives and optimization strategy used to jointly preserve segmentation fidelity and improve anomaly rejection.

### 3.1. Problem Formulation

The standard closed-set medical image segmentation task aims to learn a mapping f:X→Y, where $X\subset R^{H\times W\times 3}$ represents the input image space and $Y\subset {\left\{0,1\right\}}^{H\times W}$ represents the binary mask space indicating the presence of a lesion. However, this formulation assumes that all test samples originate from the same marginal distribution $P_{in}\left(X\right)$ observed during training. In real-world colonoscopy, a model frequently encounters out-of-distribution (OOD) artifacts, denoted by a distribution $P_{out}\left(X\right)$, such as surgical snares, biopsy forceps, or clips.

To ensure clinical safety, an open-set segmentation model must not only perform the dense prediction task f(x) for in-distribution data but also compute an anomaly score S(x) to reliably reject samples from $P_{out}\left(X\right)$. We formulate PolypSAM-Open as a joint optimization problem. We map the input *x* into a latent embedding space $Z\subset R^{d}$ via an encoder $E_{\theta }$ and simultaneously train a mask decoder $D_{\phi }$ and a prototype-based anomaly detector. The objective is to maximize segmentation fidelity for known polyps while strictly separating the structural latent representations of $P_{in}$ and $P_{out}$ around a dynamically learned prototype.

### 3.2. Datasets and Preprocessing

**Figure 3:**
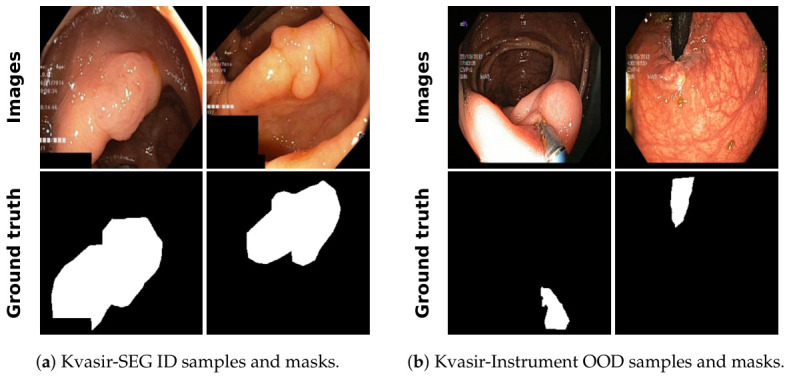
**Side-by-side examples from the study datasets.** (**a**): in-distribution Kvasir-SEG colonoscopy frames with expert polyp masks used for model development. (**b**): out-of-distribution Kvasir-Instrument frames with expert instrument masks used only for zero-shot OOD evaluation. ID: in-distribution; OOD: out-of-distribution.

We utilized the publicly available Kvasir-SEG dataset [29] as our primary cohort. This rigorously annotated collection consists of 1000 colonoscopy images. We partitioned this dataset into 880 images for training and 120 images for ID validation and testing. To enforce a true zero-shot Open-Set Learning strategy, we engineered an 85/15 data split during the training phase. Specifically, 85% of the training data consisted of authentic polyps, while the remaining 15% was replaced with simulated high-frequency Gaussian noise. This synthetic noise served to mathematically define the out-of-distribution rejection margin within the latent space. The ground truth annotations, manually delineated by medical experts, were exported to generate binary masks (Figure 3a). Bounding box prompts, essential for guiding the foundation model, were dynamically derived from the spatial extremities of these binary masks. Notably, the dataset authors preemptively masked the endoscope position marking probe (ScopeGuide, Olympus, Tokyo, Japan) with black boxes to remove superfluous visual information.

Crucially, to evaluate the zero-shot open-set robustness, we utilized the external Kvasir-Instrument dataset [6] strictly for unseen evaluation. This collection contains 590 images of various diagnostic and therapeutic surgical tools utilized during gastrointestinal endoscopy, such as snares, balloons, and biopsy forceps. The model never encountered these artifacts during the training phase. The original images exhibit variable resolutions ranging from 720×576 to 1280×1024 pixels. To ensure high-quality ground truth, the dataset authors employed a rigorous three-step annotation protocol: initial labeling by experienced research assistants, verification by an expert gastroenterologist, and final validation of the incorporated changes. Representative samples of these clinical artifacts and their corresponding masks are visualized in Figure 3b.

To ensure computational consistency across the foundation model architecture, all images and masks from both datasets were resized to a standard resolution of 256×256 pixels. We applied nearest-neighbor interpolation during mask resizing to strictly preserve the discrete binary boundaries. For the OOD instrument samples, the binary masks corresponding to the tools were used to generate bounding box prompts, simulating a scenario where a standard detection pipeline feeds an artifact into the segmentation model.

### 3.3. PolypSAM-Open Architecture and Parameter Efficiency

The proposed framework builds upon MedSAM [2], a Vision-Transformer-based foundation model pre-trained on over one million medical images. The core architecture consists of an image encoder $E_{\theta }$, a prompt encoder, and a lightweight mask decoder $D_{\phi }$. To adapt this massive architecture to our specific task while maintaining extreme computational feasibility for clinical hardware, Low-Rank Adaptation (LoRA) [3] was employed.

The pre-trained weights of the image encoder were frozen and trainable rank decomposition matrices were injected strictly into the query, key, and value (qkv) projection layers of every multi-head attention block. The LoRA hyperparameters were constrained to a rank of r=4, a scaling alpha of α=32, and a dropout probability of 0.05. The mask decoder $D_{\phi }$ was completely unfrozen.

This design choice preserves the rich medical-domain knowledge encoded in MedSAM’s pre-trained Vision Transformer while enabling task-specific adaptation with minimal parameter overhead. Freezing the image encoder and restricting LoRA updates exclusively to the qkv projections prevents catastrophic forgetting on the relatively small Kvasir-SEG dataset while retaining the model’s 1-million-image pre-training advantage [3,11,15]. The mask decoder is left unfrozen because it is lightweight and benefits from full gradient flow for precise polyp boundary refinement. The hyperparameters r=4, α=32, and dropout 0.05 follow established practices in parameter-efficient SAM adaptations for endoscopy [11,14,15], striking an optimal trade-off between adaptation capacity, training stability, and generalization on limited medical data.

This strategy is highly parameter-efficient. The framework requires training only 4,205,796 parameters out of the total 93,883,440 parameters, meaning only 4.48% of the network is updated, critical for clinical deployment where GPU memory, training time, and inference latency must remain practical.

To enable Open-Set Learning (OSL), a custom prototype mapping head was attached to the output of the image encoder. The spatial feature map $F\in R^{B\times 256\times 64\times 64}$ was processed through a global average pooling layer, flattened, and passed through a two-layer multi-layer perceptron with a ReLU activation. This projected the high-dimensional spatial features into a compact, robust latent vector $z\in R^{64}$. Global average pooling produces an image-level descriptor that is independent of the dense segmentation task. The 64-dimensional embedding was chosen to yield a low-dimensional manifold that supports stable Euclidean-distance computation in the contrastive margin loss, while avoiding the curse of dimensionality and excessive parameterization [26,28]. The entire head adds negligible overhead, ensuring the OSL module can run in parallel with segmentation in a single forward pass.

### 3.4. Loss Formulation and Prototype Learning

The network was optimized using a hybrid loss function combining dense segmentation objectives with representation learning. For the primary segmentation task, the MONAI [30] implementation of the Dice Cross-Entropy (DiceCE) loss was utilized:

(1) $$L_{seg}=L_{Dice}\left(\hat{y},y\right)+L_{CE}\left(\hat{y},y\right)$$

where $\hat{y}$ is the predicted probability mask and *y* is the ground truth.

For the anomaly detection task, we maintained a continuously updated known prototype $p\in R^{64}$, initialized as a zero vector. A contrastive margin loss was employed to enforce representation compactness for ID data and dispersion for OOD data. Here, $y^{ood}\in \left\{0,1\right\}$ is a binary indicator, where 1 denotes an OOD sample. The OSL loss for a batch of size *N* is defined as:

(2) $$L_{OSL}=\frac{1}{N}\sum_{i=1}^{N}\left[\left(1-y_{i}^{ood}\right){∥z_{i}-p∥}_{2}^{2}+y_{i}^{ood}\max{\left(0,m-{∥z_{i}-p∥}_{2}\right)}^{2}\right]$$

where m=2.0 is the predefined margin distance. The total training objective is:

(3) $$L_{total}=L_{seg}+\lambda_{OSL}L_{OSL}$$

where $\lambda_{OSL}=0.1$ controls the regularizing strength of the anomaly detection module.

During training, the known prototype *p* was not learned via backpropagation. Instead, it was updated using an Exponential Moving Average (EMA) at the end of each training epoch, using the mean of the in-distribution latent vectors aggregated over that epoch:

(4) $$p^{\left(e\right)}=\gamma \cdot p^{\left(e-1\right)}+\left(1-\gamma \right)\cdot {\bar{z}}_{ID}^{\left(e\right)}$$

where γ=0.9 is the EMA decay, *e* indexes the training epoch, and ${\bar{z}}_{ID}^{\left(e\right)}$ is the mean in-distribution latent vector aggregated over epoch *e* (the average of the per-batch in-distribution means). Throughout, *N* denotes the batch size and $N_{ID}$ the number of in-distribution samples within a batch, so that $N_{ID}\leq N$.

The open-set hyperparameters were fixed a priori from established practice and design considerations rather than tuned on the evaluation data, which avoids optimistic bias from test-set selection. The margin was set to m=2.0, a standard value for contrastive margin objectives; notably, the framework is not sensitive to its precise value, because detection at deployment relies on a threshold τ calibrated on the in-distribution distribution (Section 4.3) rather than on *m* itself, which serves only as a geometry-shaping target for the synthetic-noise proxy during training. The OSL weighting coefficient was set to $\lambda_{OSL}=0.1$ so that the auxiliary open-set objective remains subordinate to the primary segmentation loss; the preservation of segmentation fidelity relative to the ablation (Dice 0.9728 versus 0.9723) confirms that this weighting does not compromise the primary task. The prototype dimension (64) and EMA decay (γ=0.9) follow common choices for low-dimensional metric embeddings and moving-average prototype estimation, respectively, balancing representational capacity against stable Euclidean-distance computation. A systematic sensitivity analysis of these hyperparameters is a direction for future work. The comprehensive training procedure for PolypSAM-Open is delineated in Algorithm 1.
**Algorithm 1** PolypSAM-Open Training Pipeline (OOD Adaptation)**Require:** Pre-trained MedSAM weights, Kvasir-SEG ID dataset (polyps), synthetic high-frequency Gaussian noise (OOD training proxy).
 1:**Initialize Model:** Freeze ViT image encoder; inject LoRA matrices into qkv projections; unfreeze mask decoder. 2:**Initialize OSL:** Initialize prototype head weights. Set known prototype $p\leftarrow 0\in R^{64}$. 3:**Set Hyperparameters:** $\lambda_{OSL}=0.1$, EMA decay γ=0.9, margin m=2.0, learning rate $\eta =10^{-4}$. 4:**for** epoch e=1 **to** 10 **do** 5:   Initialize epoch accumulator M←[] for per-batch in-distribution means. 6:   **for** each mixed batch of *N* samples $\left(x_{i},y_{i},{\mathrm{bbox}}_{i},y_{i}^{ood}\right)$ **do** 7:    **Forward Pass:** 8:    Predict mask probabilities ${\hat{y}}_{i}$ via MedSAM decoder. 9:    Extract latent vectors $z_{i}\in R^{64}$ via prototype head.10:    **Loss Computation:**11:    $L_{seg}\leftarrow \frac{1}{N}\sum_{i=1}^{N}\left[L_{Dice}\left({\hat{y}}_{i},y_{i}\right)+L_{CE}\left({\hat{y}}_{i},y_{i}\right)\right]$12:    $d_{i}\leftarrow {∥z_{i}-p∥}_{2}$13:    $L_{OSL}\leftarrow \frac{1}{N}\sum_{i=1}^{N}\left[\left(1-y_{i}^{ood}\right)d_{i}^{2}+y_{i}^{ood}\max{\left(0,m-d_{i}\right)}^{2}\right]$14:    $L_{total}\leftarrow L_{seg}+\lambda_{OSL}L_{OSL}$15:    **Backward Pass:**16:    Compute gradients of $L_{total}$ using mixed-precision.17:    Update LoRA matrices, mask decoder, and prototype head via AdamW.18:    **Accumulate ID statistics:** append the batch in-distribution mean $\frac{1}{N_{ID}}\sum_{\left\{i|y_{i}^{ood}=0\right\}}z_{i}$ to M.19:   **end for**20:   **Prototype Update (once per epoch, ID only):**21:   ${\bar{z}}_{ID}^{\left(e\right)}\leftarrow \mathrm{mean}\left(M\right)$22:   $p\leftarrow \gamma \cdot p+\left(1-\gamma \right)\cdot {\bar{z}}_{ID}^{\left(e\right)}$23:   Update learning rate η via Cosine Annealing schedule.24:**end for**


### 3.5. Experimental Setup and Ablation

All models were implemented in PyTorch [31] (Version: 2.9.0+cu126) and trained in an NVIDIA GPU environment. To rigorously isolate the impact of the OSL module, we trained an ablation model designated as MedSAM-LoRA. Both MedSAM-LoRA and PolypSAM-Open share an identical MedSAM backbone and an identical LoRA configuration (r=4, α=32, dropout 0.05), differing solely in the presence or absence of the OSL module ($\lambda_{OSL}=0$ for MedSAM-LoRA versus $\lambda_{OSL}=0.1$ for PolypSAM-Open) and in the proportion of authentic training data used (100% authentic polyps for MedSAM-LoRA versus 85% authentic polyps plus 15% synthetic noise for PolypSAM-Open). All other optimizer settings and random seeds were held constant.

A key design choice in PolypSAM-Open is the use of high-frequency Gaussian noise as the sole OOD training signal, rather than any authentic surgical artifact. This choice is grounded in a principled geometric argument about the latent space. The OSL module’s objective is not to memorize the appearance of specific anomalies, but to learn a compact, well-separated representation of the in-distribution polyp manifold. The contrastive margin loss operates through two complementary forces: an attraction term that pulls authentic polyp embeddings toward the dynamically updated prototype, and a repulsion term, applied to the synthetic-noise proxy, that supplies the outward contrastive pressure needed to prevent representational collapse and to establish a consistent direction of separation away from the prototype. The predefined margin m=2.0 is therefore best understood as a geometry-shaping target imposed on the noise proxy during training, rather than as the operating threshold used for detection at deployment; its role is to drive the optimizer to carve out an embedding in which non-polyp content is pushed away from a tightly concentrated polyp cluster. The mechanism by which this behavior generalizes to unseen authentic instruments is in-distribution compaction rather than literal margin transfer. Because the attraction term constrains the polyp cluster to an extremely small radius, even a modest absolute displacement from the prototype is highly anomalous in standardized terms. An unseen input that does not lie on the polyp manifold, such as a surgical instrument, therefore need not be repelled as far as the noise proxy to be rejected; it need only fall outside the compact polyp cluster. As quantified in Section 4.3, authentic instruments lie many in-distribution standard deviations from the prototype, despite residing substantially closer to it than the synthetic noise, and a threshold calibrated on the in-distribution distribution alone separates them reliably in a true zero-shot manner. This reasoning aligns directly with supervised contrastive learning [32]: what matters is the relative geometry of attraction and repulsion in embedding space, not the specific visual content of the repelled samples.

### 3.6. Evaluation Metrics and Latent Space Distance

We assessed in-distribution segmentation performance using standard clinical metrics, including the Dice Similarity Coefficient, mean Intersection over Union (mIoU), Sensitivity (Recall), Specificity, Positive Predictive Value (PPV), and Negative Predictive Value (NPV). These pixel-level metrics are mathematically defined based on the total counts of True Positive (TP), True Negative (TN), False Positive (FP), and False Negative (FN) predictions across the segmentation mask:

(5) $$\begin{array}{ccc} & \mathrm{Dice} & =\frac{2\cdot TP}{2\cdot TP+FP+FN} \end{array}$$

(6) $$\begin{array}{ccc} & \mathrm{mIoU} & =\frac{TP}{TP+FP+FN} \end{array}$$

(7) $$\begin{array}{cc} \mathrm{Sensitivity} & =\frac{TP}{TP+FN} \end{array}$$

(8) $$\begin{array}{cc} \mathrm{Specificity} & =\frac{TN}{TN+FP} \end{array}$$

(9) $$\begin{array}{ccc} & \mathrm{PPV} & =\frac{TP}{TP+FP} \end{array}$$

(10) $$\begin{array}{ccc} & \mathrm{NPV} & =\frac{TN}{TN+FN} \end{array}$$

To evaluate OOD robustness during inference, we replaced standard predictive entropy with a direct structural metric termed Latent Space Distance. For any given query image, the extracted latent vector $z_{query}$ is compared against the globally learned polyp prototype *p*. The anomaly score S(x) is defined simply as the $L_{2}$ norm distance: $S\left(x\right)={∥z_{query}-p∥}_{2}$. OOD detection performance was then quantified using the Area Under the Receiver Operating Characteristic Curve (AUROC), treating ID samples as the negative class and authentic surgical instruments as the positive class. Formally, AUROC is defined as:

(11) $$\mathrm{AUROC}=\int_{0}^{1}\mathrm{TPR}\left(u\right)\;d\mathrm{FPR}\left(u\right)$$

computed by sweeping thresholds over S(x). Statistical significance was determined using the Wilcoxon Signed-Rank test for segmentation metrics and a 1000-iteration bootstrap test for AUROC comparisons.

## 4. Results

To comprehensively evaluate the PolypSAM-Open framework, we benchmarked its performance against the Zero-Shot MedSAM baseline. Subsequently, we conducted a rigorous ablation study by comparing our proposed model to the MedSAM-LoRA ablation ($\lambda_{OSL}=0.0$) to isolate the impact of explicit latent space structuring. Finally, we compared our results against state-of-the-art frameworks.

### 4.1. Quantitative Evaluation

The primary objective of a clinical segmentation model is to maintain clinically acceptable accuracy on known pathological structures while ensuring operational safety. Both fine-tuned architectures significantly outperformed the Zero-Shot MedSAM baseline across all pixel-level metrics. As detailed in Table 2, PolypSAM-Open achieved a high-fidelity Dice Similarity Coefficient of 0.9728 and an mIoU of 0.9482 on the in-distribution Kvasir-SEG test cohort, alongside its robust zero-shot OOD detection capabilities.

**Figure 4:**
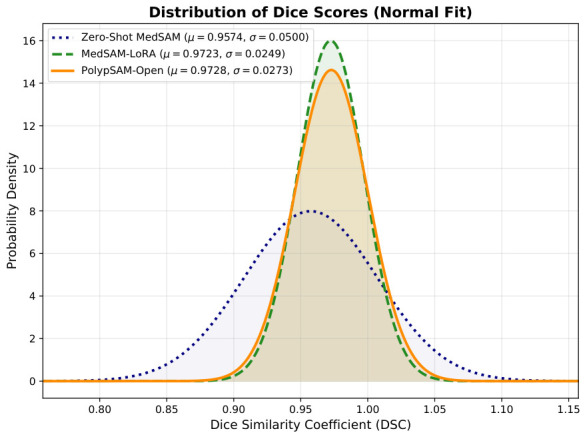
Distribution of Dice Similarity Coefficient scores (Normal Fit). The probability density functions illustrate the segmentation performance spread across the three evaluated models. Both fine-tuned architectures exhibit a significantly tighter variance and higher mean compared to the zero-shot baseline. The mathematical overlap between MedSAM-LoRA and PolypSAM-Open validates the principle of safety without clinical sacrifice. MedSAM: Medical Segment Anything Model; LoRA: Low-Rank Adaptation.

**Figure 5:**
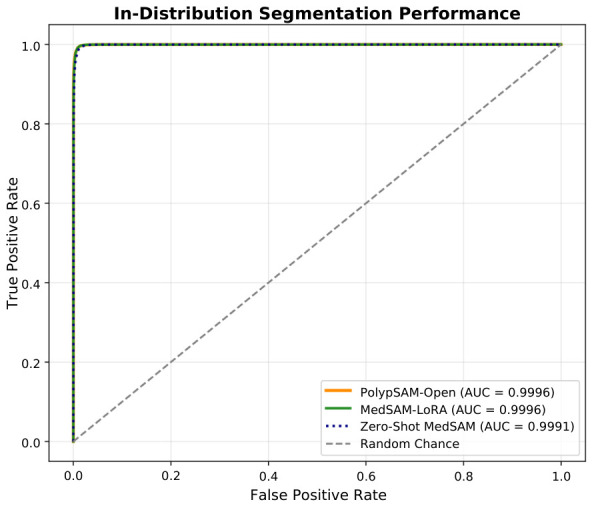
In-distribution segmentation receiver operating characteristic curves. All three models demonstrate near-perfect pixel-level classification capabilities on the Kvasir-SEG test cohort, achieving an Area Under the Curve of approximately 0.999. ROC: Receiver Operating Characteristic; AUC: Area Under the Curve.

The most compelling finding emerges when comparing the proposed PolypSAM-Open to the ablation model. The ablation model possessed the significant advantage of training exclusively on 100% authentic clinical polyps. In contrast, the proposed model dedicated 15% of its training batches strictly to simulated noise to learn its structural rejection margin. Despite this handicap, PolypSAM-Open maintained equivalent clinical efficacy, achieving a Dice score of 0.9728 compared to 0.9723 (p=0.0807), a statistically non-significant difference confirming that no clinical precision was sacrificed. Figure 4 illustrates this equivalence via the normal fit distribution of the Dice scores. Furthermore, evaluating the models exclusively on the in-distribution pixel classification task resulted in a near-perfect AUROC of approximately 0.999 for all models (Figure 5), confirming the baseline pixel thresholds remained intact.

**Figure 6:**
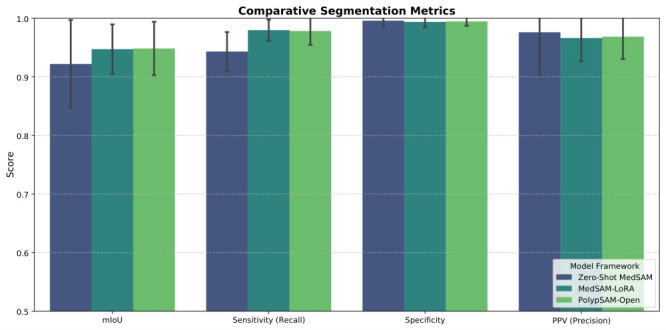
Comparative in-distribution segmentation metrics. The grouped bar chart visualizes mean Intersection over Union, Sensitivity (Recall), Specificity, and Positive Predictive Value (Precision). PolypSAM-Open successfully maintains equivalent high-fidelity segmentation metrics relative to the ablation model despite its restricted exposure to authentic training data. IoU: Intersection over Union; PPV: Positive Predictive Value.

As illustrated in Figure 6, these results indicate that the contrastive margin loss imposed by the OSL module acts as an effective regularizer. It improves separation from out-of-distribution artifacts while maintaining segmentation utility. A statistical breakdown across evaluated metrics is provided in Table 3.

### 4.2. Ablation Study: The Impact of Open-Set Learning

The primary motivation of our framework is to improve safety by reliably flagging surgical instruments. As detailed in Table 2, standard parameter-efficient fine-tuning showed limited OOD discrimination in this setting. The MedSAM-LoRA ablation model, relying solely on LoRA, achieved an OOD AUROC of 0.4263 on the unseen Kvasir-Instrument dataset. An AUROC below 0.50 indicates that the standard fine-tuned model ranked OOD samples poorly relative to in-distribution samples.

**Figure 7:**
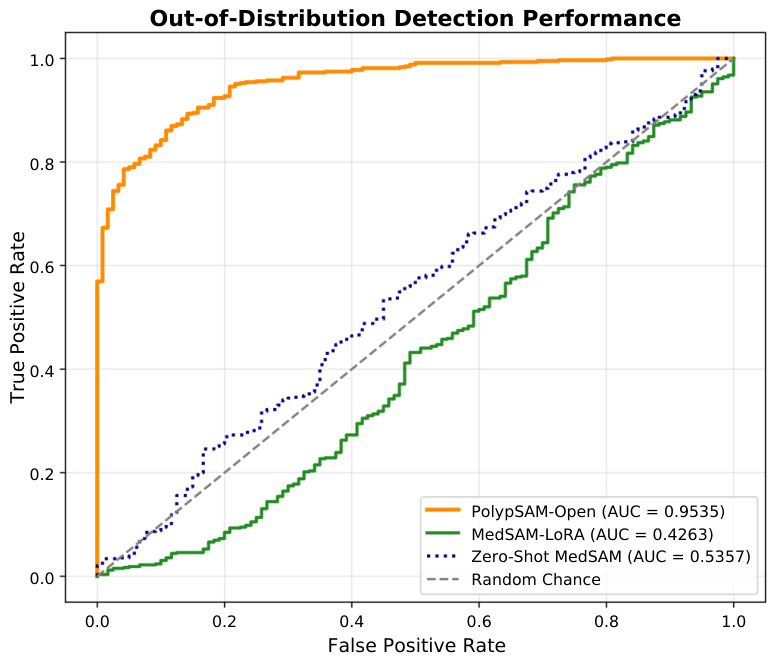
Out-of-distribution anomaly detection performance. The receiver operating characteristic curve evaluates the Latent Space Distance metric on unseen authentic surgical instruments. The ablation model (0.4263) performs below random ranking for OOD discrimination in this setting. PolypSAM-Open (0.9535) provides substantially improved separation between OOD artifacts and the in-distribution clinical data. OOD: out-of-distribution; ROC: Receiver Operating Characteristic; AUROC: Area Under the Receiver Operating Characteristic Curve; MedSAM: Medical Segment Anything Model; LoRA: Low-Rank Adaptation.

Using Latent Space Distance to evaluate the structured embedding space, PolypSAM-Open identified 590 unseen authentic surgical instruments with a zero-shot OOD AUROC of 0.9535 (Figure 7). A 1000-iteration bootstrap test indicated a statistically significant AUROC improvement over the ablation model (p<0.001).

**Figure 8:**
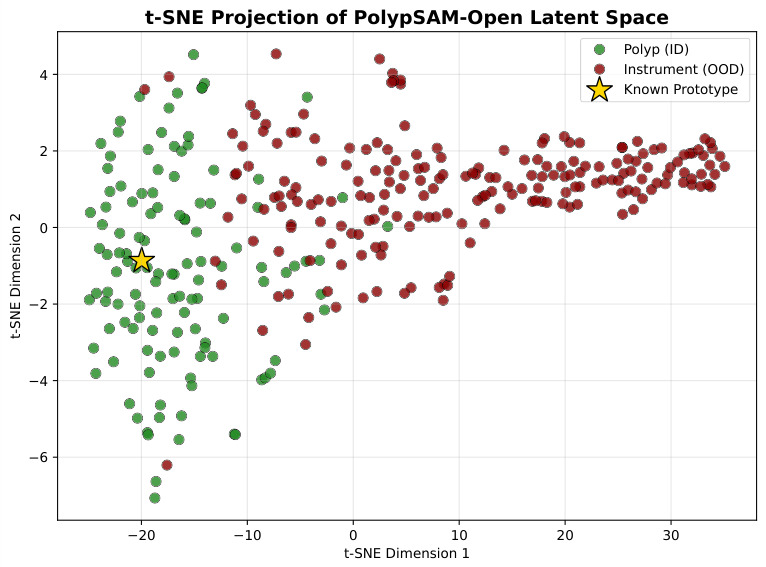
t-SNE Projection of PolypSAM-Open Latent Space. The visualization reveals the explicit structural sculpting achieved by the Open-Set Learning module. In-distribution polyp features (green) cluster tightly around the learned known prototype (yellow star), while out-of-distribution surgical instruments (red) lie outside this compact in-distribution cluster, enabling reliable thresholding via Latent Space Distance. The spread distribution of OOD points is expected: the detection mechanism operates on each sample’s individual distance from the ID prototype, not on OOD intra-cluster coherence. t-SNE: t-distributed Stochastic Neighbor Embedding; OOD: out-of-distribution; ID: in-distribution.

**Figure 9:**
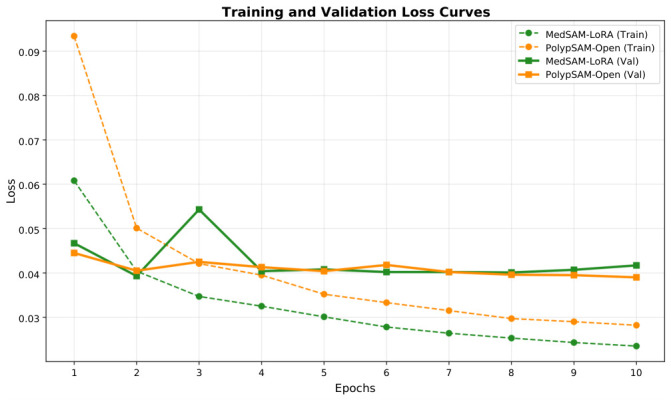
Training and validation loss curves. The plot tracks the convergence of MedSAM-LoRA and the proposed PolypSAM-Open over 10 epochs, demonstrating stable optimization for both frameworks despite the complex dual-objective loss in the proposed model. MedSAM: Medical Segment Anything Model; LoRA: Low-Rank Adaptation.

We visualized this structural divergence using a t-Distributed Stochastic Neighbor Embedding (t-SNE) projection of the PolypSAM-Open latent space (Figure 8). The projection clearly demonstrates how the OSL module successfully clusters in-distribution polyp features near the learned prototype. It is important to note that the OOD instrument points appear spatially spread rather than forming a tight counter-cluster. This behavior is expected and inconsequential to the method’s validity: the detection mechanism relies exclusively on each sample’s Euclidean distance from the ID prototype, not on OOD clustering. Since all that is required for reliable detection is that OOD representations fall outside the compact in-distribution cluster, that is, beyond the in-distribution-calibrated threshold τ (Section 4.3), their distribution relative to one another is irrelevant. Training stability was equivalent for both models, as verified through the tightly converging training and validation loss curves (Figure 9).

### 4.3. Open-Set Scoring Analysis: Scoring Functions, Operating Points, and Latent Geometry

To further characterize the open-set behavior of PolypSAM-Open, we conducted three additional analyses on the trained proposed model without any retraining: (i) a comparison of the proposed Latent Space Distance against common alternative out-of-distribution (OOD) scoring functions, (ii) a calibration of the safety-flag decision threshold to clinically interpretable operating points, and (iii) a quantitative examination of the latent-space geometry underlying zero-shot transfer from the synthetic-noise proxy to authentic instruments.

We first compared Latent Space Distance with three widely used OOD scoring functions computed on the same model: Maximum Softmax Probability (MSP), predictive entropy, and the energy score. To isolate the scoring function as the only variable, all four scores were derived from a single forward pass of the identical PolypSAM-Open model; the three baseline scores are computed from the predicted mask logits, whereas Latent Space Distance is computed from the prototype-head embedding. As shown in Table 4, the mask-based scores were largely uninformative for surgical-instrument detection (MSP AUROC 0.5074, predictive entropy 0.5234, energy 0.6785), whereas Latent Space Distance achieved 0.9535. This disparity is consistent with the structure of the failure mode: surgical instruments possess sharp, well-defined edges that the decoder segments with high confidence, yielding low-entropy, high-confidence masks that mask-based uncertainty cannot distinguish from genuine polyps. Operating on the semantic embedding rather than the decoder output, Latent Space Distance remains sensitive to this distributional shift even when the predicted mask appears geometrically coherent.

Although AUROC summarizes detection performance across all thresholds, clinical deployment requires committing to a single decision boundary. We therefore calibrated the safety-flag threshold on the in-distribution distribution alone, selecting the value that retains a prescribed in-distribution specificity (equivalently, a fixed false-alarm budget) without reference to any OOD sample. Table 5 reports sensitivity, specificity, precision, and F1 at three such operating points, together with the Youden-optimal point. At a 95% in-distribution specificity budget, the flag detects 78.98% of unseen instruments; relaxing the budget to 90% raises instrument sensitivity to 84.24%, and the Youden-optimal threshold yields a balanced 86.78% sensitivity at 88.33% specificity. This trade-off makes the balance between alert fatigue and missed detections explicit and tunable to site-specific tolerances. We note that the reported precision and F1 reflect the composition of the evaluation set (120 in-distribution versus 590 instrument images) rather than the prevalence of instrument frames in routine colonoscopy, and would scale with the operational base rate.

Finally, we examined the latent geometry that enables zero-shot transfer from the synthetic-noise training proxy to authentic instruments. Table 6 reports the Latent Space Distance of each group from the learned prototype. Two observations clarify the operative mechanism. First, the in-distribution polyp embeddings form an extremely compact cluster (mean distance 0.0207, standard deviation 0.0076), confirming that the attraction term drives strong representational concentration. Second, although the synthetic noise is repelled far beyond the training margin (mean distance 2.2889, with 99.17% of samples exceeding m=2.0), authentic instruments reside much closer to the prototype in absolute terms (mean distance 0.1663) and do not approach the noise rejection zone. Detection nonetheless succeeds because the polyp cluster is so concentrated that instruments lie, on average, 19.27 in-distribution standard deviations from the prototype. The operative mechanism is therefore in-distribution compaction rather than literal margin transfer: the training margin m=2.0 is a geometry-shaping target imposed on the noise proxy, whereas the deployment threshold calibrated on in-distribution data alone (τ=0.0355 at 95% specificity) is more than an order of magnitude smaller and is the boundary that actually separates instruments from polyps. Consistent with this account, the same model cleanly separates polyps from both the synthetic noise (AUROC 1.0000) and the unseen authentic instruments (AUROC 0.9535), indicating that the learned representation rejects non-polyp content broadly rather than memorizing the appearance of the noise proxy.

### 4.4. Qualitative Visual Analysis

**Figure 10:**
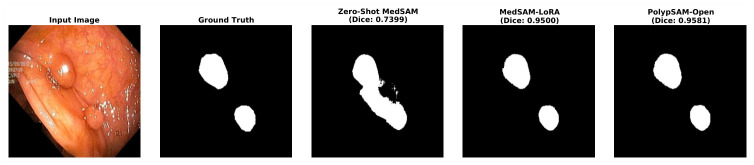
Qualitative comparison of colorectal polyp segmentation. The visualization compares model predictions against the expert-annotated ground truth. PolypSAM-Open successfully conforms to complex morphological boundaries, producing a tighter, more precise segmentation mask relative to both the zero-shot baseline and MedSAM-LoRA. MedSAM: Medical Segment Anything Model; LoRA: Low-Rank Adaptation.

Qualitative assessment corroborates the quantitative findings, illustrating the superior boundary fidelity of the proposed network. Figure 10 provides a progressive visual comparison across the evaluated models. The Zero-Shot MedSAM baseline frequently exhibits severe boundary under-segmentation. While the standard MedSAM-LoRA ablation model significantly improves upon the baseline, it occasionally struggles with complex morphological borders. In contrast, PolypSAM-Open not only avoids under-segmentation but actively refines the most challenging local boundaries, producing a highly conformal mask that tightly adheres to the target lesion. Visually, this confirms that the structural regularizer imposed by the open-set learning module actively enhances pixel-level predictive quality, aligning with the improved quantitative Dice metrics.

### 4.5. Comparison with the State of the Art

To contextualize performance, we compared PolypSAM-Open against twelve recent segmentation frameworks evaluated on the Kvasir-SEG dataset. These include pure convolutional networks (such as ResUNet++, Focus U-Net, and ResSegNet++), hybrid architectures (PolypMoE), and parameter-efficient adaptations of foundation models (FCSAM, PSF-SAM, and BiSeg-SAM).

As shown in Table 7, PolypSAM-Open achieved the highest Dice Similarity Coefficient (0.9728) among the evaluated methods. The method exceeded the highest-performing purely convolutional comparator, ResSegNet++ (0.9457), and yielded higher Dice than other SAM-based adaptations, including PSF-SAM (0.9393) and FCSAM (0.9370). These results suggest that integrating prototype learning with LoRA can improve OOD robustness while maintaining strong pixel-wise segmentation performance.

### 4.6. External Generalizability

To assess external generalizability, we evaluated two independent datasets, ETIS-LaribPolypDB [42] and CVC-ClinicDB [43]. As shown in Table 8, the proposed method showed consistent performance across these unseen cohorts. On CVC-ClinicDB, PolypSAM-Open achieved slightly higher boundary Dice (0.9386 versus 0.9364) and mIoU (0.8924 versus 0.8894) than the ablation model. On ETIS-LaribPolypDB, PolypSAM-Open and the ablation model were closely matched (Dice 0.9301 versus 0.9305). In-distribution AUROC remained high (approximately 0.999) across external cohorts, suggesting that the decision behavior is stable across sites. The modest reduction in Dice on the external cohorts relative to the in-domain Kvasir-SEG test set (approximately 0.93 versus 0.97) reflects cross-dataset domain shift rather than prototype fixation or overfitting introduced by the open-set module. This is evidenced by the ablation model, which contains no prototype-based open-set component yet exhibits an essentially identical reduction (ETIS-LaribPolypDB Dice 0.9305; CVC-ClinicDB Dice 0.9364), closely tracking the proposed model (0.9301 and 0.9386, respectively). Because a model without the prototype mechanism degrades to the same degree, the gap is attributable to differences in imaging characteristics across centers, which affect both models equally, and not to the learned prototype overfitting to the training cohort.

### 4.7. Hardware and Computational Profiling

For a clinical decision support system to be viable during intra-procedural colonoscopy, it must operate with minimal latency. As shown in Table 9, we profiled the computational overhead of our proposed framework against the baselines, measuring total parameters, floating-point operations (GFLOPs), and inference latency.

PolypSAM-Open performs inference in 165.594 ms per image (6.04 frames per second) on the profiled hardware. Because the open-set scoring head reuses the image embedding already computed for segmentation, it requires no additional encoder pass, and the three models are comparable in latency (165.594 to 173.639 ms), with the differences falling within run-to-run measurement variance. This indicates that the open-set capability adds no meaningful computational overhead relative to the underlying MedSAM forward. This latency must, however, be interpreted against the demands of live endoscopy. Standard endoscopic video is acquired at 25 to 30 frames per second (approximately 33 to 40 ms per frame), so a per-image latency of roughly 166 ms corresponds to about five frames at 30 frames per second. Consequently, PolypSAM-Open in its current form cannot process every consecutive video frame, and if its mask and safety-flag outputs were rendered as a live overlay during rapid endoscope motion, they would lag the displayed scene and could become spatially misaligned with the current field of view. The framework is therefore positioned as a decision-support tool that operates over a short analysis window, for example when the endoscopist holds the view on a region of interest, rather than as a frame-locked real-time overlay [44]. Reducing latency toward video frame rate, and incorporating temporal smoothing to maintain frame-to-frame consistency of the safety flag, are important directions for clinical translation and are considered further in Section 5.

## 5. Discussion

The deployment of deep learning models in colonoscopy represents a significant step toward automating polyp detection and standardizing clinical quality indicators [44,45,46]. However, the transition from closed-set laboratory benchmarks to highly variable clinical environments exposes critical safety vulnerabilities. Our proposed framework, PolypSAM-Open, addresses these vulnerabilities by embedding open-set robustness directly into a parameter-efficient foundation model. The results support our central hypothesis of improved zero-shot rejection of out-of-distribution artifacts without an observable reduction in segmentation performance. PolypSAM-Open substantially improves out-of-distribution (OOD) anomaly detection on unseen authentic surgical instruments while maintaining segmentation fidelity comparable to standard fine-tuning.

### 5.1. Limitations of Standard Parameter-Efficient Fine-Tuning in Open-World Clinical Settings

Historically, medical image segmentation has relied on specialized convolutional architectures such as U-Net [47], Attention U-Net [48], and TransUNet [49]. The recent advent of the Segment Anything Model (SAM) [50] and its clinical adaptations [51] has shifted this paradigm. While these models possess vast generalization capabilities, they require careful tuning to handle specific medical modalities [52,53]. Standard parameter-efficient techniques, such as adapter tuning [54] and delta tuning [55], excel at domain adaptation but inherently assume that all inference data will belong to the target distribution.

A primary motivation of this study was to evaluate the clinical readiness of foundation models adapted with standard parameter-efficient techniques. The ablation analysis identified a safety limitation of standard Low-Rank Adaptation (LoRA). On 590 unseen authentic surgical instruments from Kvasir-Instrument, the MedSAM-LoRA ablation model achieved an OOD AUROC of 0.4263. Because this value is below 0.50, the model performed worse than random ranking for OOD discrimination in this setting. In qualitative examples, the fine-tuned model often segmented surgical tools as polyps. In clinical deployment, such false positives may reduce clinician trust and interfere with workflow [56]. These findings indicate that parameter-efficient fine-tuning alone may be insufficient for robust open-world endoscopy settings without an explicit anomaly-detection mechanism.

Documenting this behavior is relevant to responsible medical-AI translation. The observation that a standard fine-tuned model attains an OOD AUROC of 0.4263 while misclassifying surgical instruments as polyps highlights a potentially important failure mode. Transparent reporting of these failure modes supports safety-focused model development and evaluation. PolypSAM-Open addresses this failure mode by quantifying it and introducing a structural mitigation strategy.

### 5.2. Automation Bias, Clinical Trust, and the Role of Uncertainty Signaling

The deployment of AI decision-support tools in colonoscopy creates conditions favorable to automation bias. Endoscopists operating under time pressure during screening procedures may default to accepting AI outputs, particularly when those outputs appear geometrically precise and high-confidence. The ablation analysis in this study documents a concrete instance of this risk: a standard LoRA-fine-tuned model achieves an OOD AUROC of 0.4263 on surgical instruments, meaning it ranks instruments as more in-distribution than actual polyps, yet its segmentation masks are visually coherent and carry no inherent uncertainty marker. A clinician with no external mechanism for detecting this failure would receive a confident, well-formed false positive. PolypSAM-Open’s Latent Space Distance score is designed to provide such an external signal. Rather than suppressing uncertainty, the framework surfaces it as an explicit, interpretable alert when the input departs from the training distribution. This design aligns with emerging recommendations for human-AI teaming in clinical environments: AI systems should not only perform their primary task accurately but should communicate the boundaries of their competence transparently [8,57]. By raising a safety flag rather than producing a potentially misleading mask, PolypSAM-Open is intended to support appropriate reliance: the clinician is informed that the AI is operating in an unvalidated regime and should apply independent judgment. We emphasize that this human-AI interaction benefit is a design rationale; it was not assessed in a clinical or user study and remains to be validated prospectively. It is worth noting that this mechanism does not require the clinician to understand the underlying latent-space geometry. The output is a binary flag (anomaly detected/not detected) accompanied by a continuous distance score for those who want granularity. This simplicity is deliberate: effective human-AI interfaces in clinical settings benefit from outputs that match clinician cognitive workflows rather than requiring interpretation of probabilistic quantities [57].

### 5.3. Latent Space Distance vs. Mask Entropy

Detecting OOD instances is a major challenge in reliable machine learning. Traditional uncertainty estimation techniques rely on computationally expensive methods such as Monte Carlo Dropout [58] or Deep Ensembles [59]. Post-hoc scoring methods like ODIN [60], Mahalanobis distance [61], and Energy-based models [62] offer lightweight alternatives but are typically optimized for image classification rather than dense pixel-level segmentation. In the specific context of endoscopy, previous anomaly detection efforts have largely focused on sparse video frames or wireless capsule endoscopy [63,64].

To resolve this vulnerability, PolypSAM-Open explicitly sculpts the latent embedding space rather than relying on standard predictive mask entropy. Traditional uncertainty metrics, such as evaluating the entropy of the final segmentation mask, frequently fail on objects like surgical tools. Because instruments such as metallic snares or forceps possess sharp, well-defined edges, foundation models inherently lock onto these boundaries with high confidence, yielding low entropy scores that mask the underlying error. This failure mode is confirmed empirically: evaluated on the same proposed model, mask-derived scores were near-chance for instrument detection (Maximum Softmax Probability AUROC 0.5074, predictive entropy 0.5234, energy 0.6785), whereas the latent-space score reached 0.9535 (Section 4.3).

By contrast, our Open-Set Learning (OSL) module operates on semantic latent representations. It learns a compact representation of the in-distribution polyp manifold, drawing on ideas from supervised contrastive learning [32] and dynamic prototype debiasing [28]. The contrastive margin loss concentrates authentic polyp embeddings tightly around the learned prototype while using the synthetic-noise proxy to supply outward separation pressure, so that unseen non-polyp inputs fall outside the compact cluster and are flagged by an in-distribution-calibrated distance threshold (Section 4.3). This design is consistent with related work in federated open-set recognition [65,66] and class-aware hyperspectral imaging [67]. Using Latent Space Distance, the model distinguishes surgical tools from polyps in representation space even when edge structure appears visually well-defined. Empirically, this is associated with improved zero-shot OOD AUROC (0.9535).

### 5.4. Clinical Implications and Trade-Offs

An important outcome of this study is that improved zero-shot safety can be obtained without measurable loss in segmentation accuracy in this dataset. The ablation model was trained on 100% authentic polyp samples, whereas PolypSAM-Open used 85% authentic polyp samples and 15% synthetic high-frequency noise to learn an anomaly rejection margin. Under this setting, PolypSAM-Open maintained segmentation performance comparable to the ablation model (Dice 0.9728 versus 0.9723, p=0.0807).

Prior work has shown that targeted optimization strategies, including knowledge distillation, architectural compression, and lightweight explainable modeling, can improve or preserve clinically relevant diagnostic behavior while substantially reducing computational overhead [68,69,70]. Consistent with this direction, the prototype margin loss in our framework encourages adherence to known anatomical boundaries while increasing separation from unexpected surgical artifacts in latent space. For decision-support deployment, such behavior may improve transparency when inputs deviate from the training distribution.

PolypSAM-Open’s safety-flag mechanism may support learning-health-system style quality workflows in endoscopy units. The Latent Space Distance score provides an interpretable real-time signal: when it exceeds the learned margin threshold, the system flags the frame as anomalous rather than producing a potentially misleading segmentation mask. This explicit alert can facilitate transparent handling of out-of-distribution inputs.

Beyond segmentation accuracy, the safety-flag mechanism has practical implications for how AI tools are integrated into endoscopic workflows. Current CADe systems typically provide a detection bounding box or overlay without communicating distributional uncertainty. PolypSAM-Open’s anomaly signal could be surfaced as a simple status indicator in the colonoscopy video feed, for example, a color-coded border distinguishing confirmed in-distribution detections from flagged anomalies. Such interface designs have been proposed in the broader human-AI interaction literature as mechanisms for calibrating clinician trust appropriately across varying levels of system confidence. Prospective evaluation of how endoscopists respond to and act upon such signals, including whether the flag reduces inappropriate polyp-labeling of surgical instruments in practice, is a priority for future work. More broadly, the safe clinical adoption of AI-assisted colonoscopy depends not only on detection performance but also on real-world implementation factors, including regulatory approval, reimbursement, medicolegal liability, automation-related deskilling, and the management of false positives, which recent work has identified as central to translating these systems into routine practice [71,72].

### 5.5. Limitations and Future Work

While evaluation on 590 authentic surgical instruments supports open-set robustness in this setting, the study focuses on static two-dimensional frames. Real-world colonoscopy is a dynamic procedure with continuous video input. A key limitation is therefore the absence of temporal context. In particular, because the safety flag is computed independently per frame with no temporal smoothing or consistency mechanism, it may toggle intermittently (“chatter”) in borderline situations, such as when a surgical instrument partially enters or exits the field of view; temporal aggregation or hysteresis to stabilize the flag across consecutive frames is therefore a necessary component of any video deployment. Future work will evaluate PolypSAM-Open on continuous video to incorporate temporal consistency and frame-to-frame anomaly tracking.

A further limitation concerns the scope of the out-of-distribution evaluation, which considered surgical instruments as the OOD class. Routine colonoscopy presents many other sources of distributional shift, including intraluminal blood, smoke from electrocautery, motion blur, specular reflections, residual stool, and illumination variation. These artifacts differ from surgical instruments in their visual and structural characteristics, and the present study does not establish how reliably the latent-space rejection mechanism flags them. Because detection operates on departure from the compact in-distribution polyp manifold rather than on instrument-specific features, we anticipate that the approach will extend to other non-polyp inputs; however, this expectation remains to be tested empirically. Systematic evaluation across a broader taxonomy of endoscopic artifacts is an important direction for establishing the full safety envelope of the framework.

An additional limitation pertains to training data provenance. Although generalizability was validated across three independent test cohorts, Kvasir-SEG, ETIS-LaribPolypDB, and CVC-ClinicDB, all training data originated from a single dataset (Kvasir-SEG). The extent to which the learned polyp prototype reflects a truly universal polyp manifold rather than the specific imaging characteristics of a single center remains an open question. Future work should explore multi-center training data to further strengthen generalizability claims and to assess whether a prototype learned from diverse acquisition conditions better separates OOD instruments from the broader distribution of polyp appearances encountered across hospital systems. Deploying the framework in prospective clinical trials will also be a critical next step to measure its real-time impact on endoscopist adenoma detection rates and workflow efficiency.

## 6. Conclusions

In this study, we introduced PolypSAM-Open, a framework for adapting a medical foundation model to colorectal polyp segmentation with explicit handling of surgical-artifact OOD inputs. Standard parameter-efficient fine-tuning yielded an OOD AUROC of 0.4263 on unseen surgical instruments, whereas the proposed model achieved 0.9535 on 590 unseen authentic tools using a rejection margin learned from synthetic noise. Despite allocating 15% of training capacity to this margin, PolypSAM-Open maintained segmentation performance comparable to the standard fine-tuned baseline (Dice 0.9728, p=0.0807 versus ablation). The method updates 4.48% of model parameters while adding negligible inference latency relative to the underlying foundation-model forward. These findings indicate that prototype-based open-set adaptation is a promising direction for potentially safer decision-support segmentation in colonoscopy and may be transferable to other medical computer-vision settings with similar OOD risks. We note, however, that this study is based entirely on retrospective, publicly available datasets; prospective clinical validation, including evaluation in live procedural settings and user studies with endoscopists, is required before any claim of improved clinical safety can be established and before routine implementation. More broadly, the results suggest that surfacing distributional uncertainty as an actionable clinical signal, rather than suppressing it within confident-looking masks, is a practical design principle for human-AI collaboration tools in procedural medicine, where automation bias poses a documented patient-safety risk.

## Figures and Tables

**Table 1:** Comparison of our proposed framework with related methods in endoscopic image analysis.

Method	Foundation Model	Parameter-Efficient	OOD Robustness	Task Focus
MedSAM [2]	Yes	No (Zero-Shot)	No	General Segmentation
PolypSAMFL [14]	Yes	Yes (LoRA)	No	Polyp Segmentation
ColonOOD [21]	No	No	Yes	Polyp Classification
SCI-OSR [27]	No	No	Yes	General OSR
**PolypSAM-Open (Ours)**	**Yes**	**Yes (LoRA)**	**Yes**	**Polyp Segmentation**

Note. Our method is the only framework combining foundation model parameter efficiency with explicit out-of-distribution robustness for pixel-level segmentation. OOD: out-of-distribution; LoRA: Low-Rank Adaptation; OSR: Open-Set Recognition; MedSAM: Medical Segment Anything Model; SAM: Segment Anything Model.

**Table 2:** Quantitative evaluation of in-distribution polyp segmentation and out-of-distribution detection on the primary Kvasir datasets.

Model	Dice	mIoU	Sensitivity	Specificity	PPV	NPV	OOD AUROC
Zero-Shot MedSAM	0.9574	0.9218	0.9431	0.9958	0.9760	0.9894	0.5357
MedSAM-LoRA (Ablation)	0.9723	0.9472	0.9796	0.9937	0.9662	0.9961	0.4263
**PolypSAM-Open (Proposed)**	**0.9728**	**0.9482**	**0.9781**	**0.9945**	**0.9684**	**0.9953**	**0.9535**

Note. PolypSAM-Open successfully resolves the critical OOD vulnerability of standard fine-tuning in a zero-shot setting, achieving a 50+ point safety gain. Despite trading 15% of authentic training data to learn the rejection margin, the proposed model maintains equivalent segmentation fidelity to the ablation (p=0.0807 for Dice). mIoU: mean Intersection over Union; PPV: Positive Predictive Value; NPV: Negative Predictive Value; OOD: out-of-distribution; AUROC: Area Under the Receiver Operating Characteristic Curve; MedSAM: Medical Segment Anything Model; LoRA: Low-Rank Adaptation.

**Table 3:** Statistical significance testing across all evaluated metrics.

Evaluation Metric	Proposed vs. Zero-Shot (*p*-Value)	Proposed vs. Ablation (*p*-Value)
Dice Similarity Coefficient	$1.16\times 10^{-15}$	$8.07\times 10^{-2}$
mean Intersection over Union (mIoU)	$1.04\times 10^{-15}$	$7.54\times 10^{-2}$
Sensitivity (Recall)	$2.64\times 10^{-19}$	$8.45\times 10^{-1}$
Specificity	$3.15\times 10^{-14}$	$3.18\times 10^{-3}$
Positive Predictive Value (PPV)	$3.77\times 10^{-14}$	$1.60\times 10^{-2}$
Negative Predictive Value (NPV)	$5.64\times 10^{-19}$	$7.16\times 10^{-1}$
In-Distribution Segmentation AUROC	<0.001	<0.001
OOD Detection AUROC	<0.001	<0.001

Note. Segmentation metrics were evaluated using the Wilcoxon Signed-Rank test. AUROC metrics were evaluated using a 1000-iteration bootstrap test. A *p*-value <0.05 is considered statistically significant. The non-significant difference in Dice (p=0.0807) and Sensitivity (p=0.8450) between the proposed and ablation models confirms that safety is achieved without clinical compromise. mIoU: mean Intersection over Union; PPV: Positive Predictive Value; NPV: Negative Predictive Value; AUROC: Area Under the Receiver Operating Characteristic Curve; OOD: out-of-distribution.

**Table 4:** Comparison of out-of-distribution scoring functions on the proposed PolypSAM-Open model (in-distribution Kvasir-SEG validation versus 590 unseen authentic Kvasir-Instrument samples). All scores are obtained from a single forward pass of the same model.

Scoring Function	Operates On	OOD AUROC
Maximum Softmax Probability (1−MSP)	Mask logits	0.5074
Predictive Entropy	Mask logits	0.5234
Energy	Mask logits	0.6785
**Latent Space Distance (proposed)**	Latent space	**0.9535**

Note. Mask-based scores are largely uninformative because surgical instruments yield confident, low-entropy masks; the latent-space score remains sensitive to the distributional shift. OOD: out-of-distribution; AUROC: Area Under the Receiver Operating Characteristic Curve; MSP: Maximum Softmax Probability.

**Table 5:** Safety-flag operating points for PolypSAM-Open. The decision threshold on the Latent Space Distance score is calibrated using the in-distribution distribution only (a fixed false-alarm budget), without reference to any out-of-distribution sample.

Operating Point	Threshold	Sensitivity	Specificity	Precision	F1
In-distribution specificity 90%	0.0319	0.8424	0.9000	0.9764	0.9045
In-distribution specificity 95%	0.0355	0.7898	0.9500	0.9873	0.8776
In-distribution specificity 99%	0.0439	0.6797	0.9833	0.9950	0.8077
Youden-optimal	0.0300	0.8678	0.8833	0.9734	0.9176

Note. Sensitivity denotes the proportion of unseen authentic instruments correctly flagged. Precision and F1 reflect the evaluation-set composition (120 in-distribution versus 590 instrument images) rather than clinical prevalence. F1: harmonic mean of precision and sensitivity.

**Table 6:** Latent Space Distance of each group from the learned in-distribution prototype, illustrating the in-distribution compaction mechanism. Distances are reported in absolute terms and in units of the in-distribution standard deviation ($\sigma_{ID}$).

Group	Mean Distance	Std. Dev.	Mean Distance ($\sigma_{\mathrm{ID}}$)	Fraction > τ
Polyp (in-distribution)	0.0207	0.0076	0.00	0.0500
Synthetic noise (training proxy)	2.2889	0.1372	300.18	1.0000
Authentic instrument (OOD)	0.1663	0.2109	19.27	0.7898

Note. τ=0.0355 is the deployment threshold at 95% in-distribution specificity (Table 5). The training margin m=2.0 is a geometry-shaping target for the noise proxy, not the deployment threshold. Authentic instruments lie far from the prototype in standardized terms despite residing closer than the noise proxy in absolute terms. ID: in-distribution; OOD: out-of-distribution; Std. Dev.: standard deviation.

**Table 7:** Comparison of PolypSAM-Open against recent methods on the Kvasir-SEG dataset.

Method	Year	Dice	mIoU
**PolypSAM-Open (Ours)**	**2026**	**0.9728**	**0.9482**
ResSegNet++ [33]	2025	0.9457	0.6680
PSF-SAM [34]	2025	0.9393	0.8997
FCSAM [35]	2024	0.937	0.885
PolySAM-Lite [15]	2026	0.9348	0.8855
PolypMoE [36]	2026	0.927	0.878
BiSeg-SAM [37]	2024	0.919	0.882
Li-SegPNet [38]	2023	0.9058	0.8800
Wang et al. [39]	2025	0.8715	0.8021
ResUNet++ [40]	2019	0.8133	0.7927
ResUNet [29]	2019	0.7878	0.7777
SP-SAM [41]	2025	0.6592	0.5574

Note. Our proposed framework achieves the highest Dice Similarity Coefficient and mean Intersection over Union scores among the evaluated methods. Comparator values are reproduced from the respective publications; because these studies may differ in data splits, preprocessing, prompt generation, and training protocols, this comparison is indicative rather than a strictly controlled benchmark. DSC: Dice Similarity Coefficient; mIoU: mean Intersection over Union; SAM: Segment Anything Model.

**Table 8:** External generalizability metrics on independent standard datasets.

Dataset	Model	Dice	mIoU	Sensitivity	Specificity	PPV	NPV	AUROC
ETIS-LaribPolypDB	MedSAM-LoRA	0.9305	0.8785	0.9318	0.9963	0.9383	0.9976	0.9994
ETIS-LaribPolypDB	**PolypSAM-Open**	**0.9301**	**0.8770**	**0.9295**	**0.9967**	**0.9370**	**0.9975**	**0.9995**
CVC-ClinicDB	MedSAM-LoRA	0.9364	0.8894	0.9428	0.9929	0.9398	0.9932	0.9982
CVC-ClinicDB	**PolypSAM-Open**	**0.9386**	**0.8924**	**0.9414**	**0.9936**	**0.9435**	**0.9929**	**0.9981**

Note. The proposed framework demonstrates a consistent and reproducible generalization profile across external datasets, confirming its reliability in diverse clinical settings. mIoU: mean Intersection over Union; PPV: Positive Predictive Value; NPV: Negative Predictive Value; AUROC: Area Under the Receiver Operating Characteristic Curve; MedSAM: Medical Segment Anything Model; LoRA: Low-Rank Adaptation.

**Table 9:** Hardware and computational profiling during model inference.

Model	Parameters (M)	GFLOPs	Latency (ms)	FPS
Zero-Shot MedSAM	93.736	743.978	173.639	5.76
MedSAM-LoRA (Ablation)	93.924	743.978	171.594	5.83
**PolypSAM-Open (Ours)**	**93.924**	**743.978**	**165.594**	**6.04**

Note. Because the open-set scoring head reuses the segmentation image embedding, no additional encoder pass is required, and all three models are comparable in latency; the differences fall within run-to-run measurement variance. M: million; GFLOPs: giga floating-point operations; ms: milliseconds; FPS: frames per second; MedSAM: Medical Segment Anything Model; LoRA: Low-Rank Adaptation.

## Data Availability

The datasets analyzed during the current study are publicly available. The Kvasir-SEG dataset utilized for in-distribution training and testing is accessible at https://datasets.simula.no/kvasir-seg [29] (20 June 2026). The Kvasir-Instrument dataset utilized for out-of-distribution evaluation is available at https://datasets.simula.no/kvasir-instrument [6] (20 June 2026). The ETIS-LaribPolypDB and CVC-ClinicDB datasets utilized for external generalizability validation are available at https://www.kaggle.com/datasets/nguyenvoquocduong/etis-laribpolypdb [42] (20 June 2026) and https://polyp.grand-challenge.org/CVCClinicDB [43] (20 June 2026), respectively. The code associated with this study is publicly available in our GitHub repository at https://github.com/umarbhasan/polypsam-open (20 June 2026).
